# Moderate Smoking

**Published:** 1914-05

**Authors:** 


**Moderate Smoking** is considered harmless by P. K. Pel of Amsterdam, our Associate Editor, Berliner Klin. Woch., No. 11, 1914.

